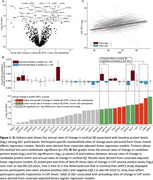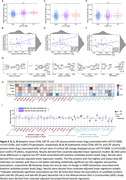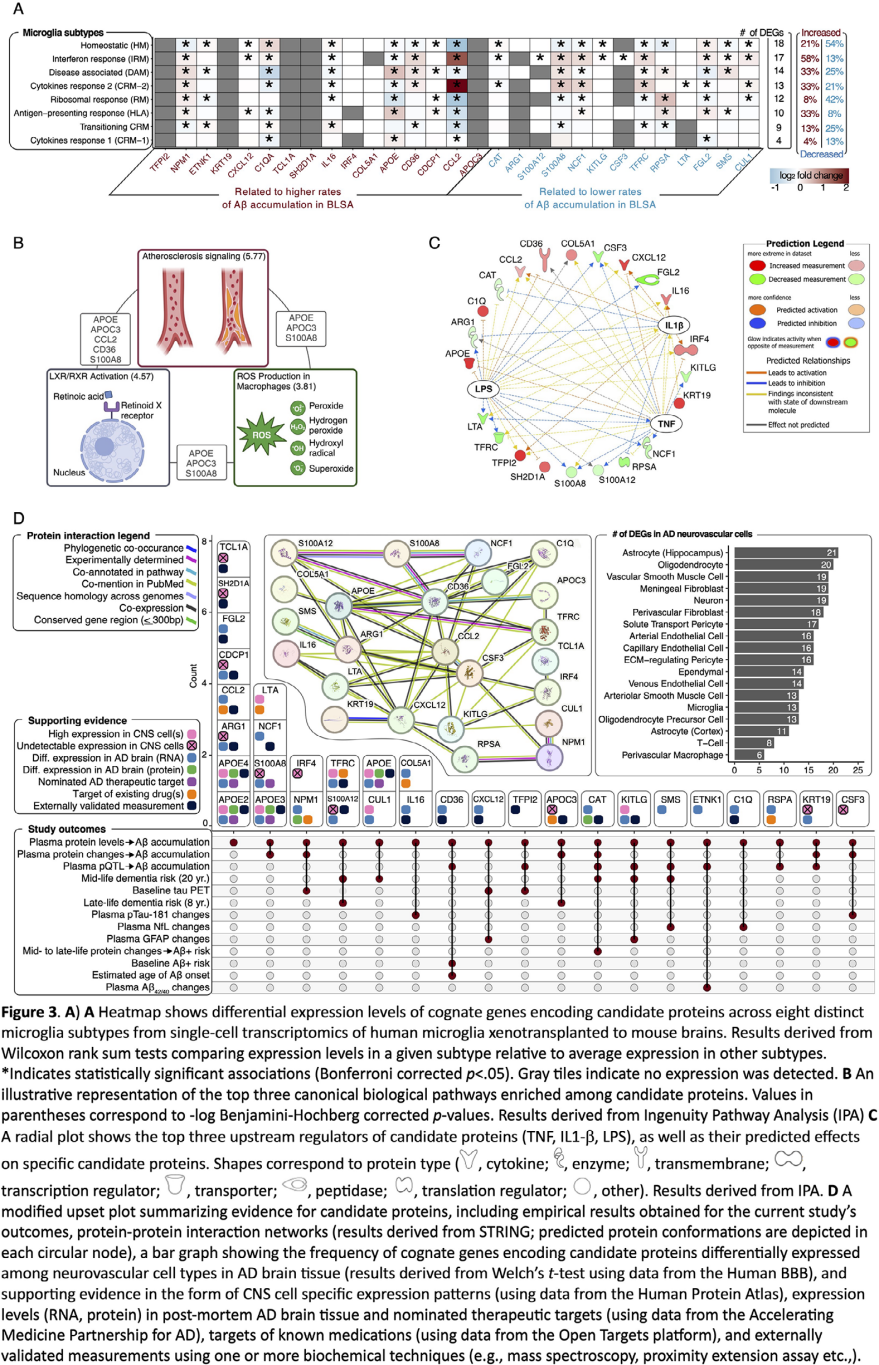# Proteome‐wide analyses identifies plasma immune regulators of amyloid‐beta progression

**DOI:** 10.1002/alz.084805

**Published:** 2025-01-03

**Authors:** Michael R. Duggan, Gabriela T Gomez, Murat Bilgel, Jingsha Chen, Nicola Fattorelli, Timothy J. Hohman, Renzo Mancuso, Jenifer Cordon, Tonnar Castellano, Mary Ellen I. Koran, Julián Candia, Alexandria Lewis, Abhay Moghekar, Nicholas J. Ashton, Przemyslaw Radoslaw Kac, Thomas K Karikari, Kaj Blennow, Henrik Zetterberg, Anna Martinez‐Muriana, Bart De Strooper, Madhav Thambisetty, Luigi Ferrucci, Rebecca F. Gottesman, Josef Coresh, Susan M. Resnick, Keenan A. Walker

**Affiliations:** ^1^ National Institute on Aging, Baltimore, MD USA; ^2^ Johns Hopkins University School of Medicine, Baltimore, MD USA; ^3^ National Institute on Aging, National Institutes of Health, Baltimore, MD USA; ^4^ Johns Hopkins Bloomberg School of Public Health, Baltimore, MD USA; ^5^ Flanders Institute for Biotechnology, Leuven Belgium; ^6^ Vanderbilt University Medical Center, Nashville, TN USA; ^7^ Flanders Institute for Biotechnology, Antwerp Belgium; ^8^ Vanderbilt Memory and Alzheimer’s Center, Institute for Medicine and Public Health, Vanderbilt University Medical Center, Nashville, TN USA; ^9^ Vanderbilt Memory & Alzheimer’s Center, Vanderbilt University Medical Center, Nashville, TN USA; ^10^ University of Gothenburg, Mölndal Sweden; ^11^ University of Gothenburg, Mölndal, Gothenburg Sweden; ^12^ Translational Gerontology Branch, National Institute on Aging, NIH, Baltimore, MD USA; ^13^ National Institute of Neurological Disorders & Stroke, Bethesda, MD USA; ^14^ Johns Hopkins University Bloomberg School of Public Health, Baltimore, MD USA; ^15^ Laboratory of Behavioral Neuroscience, National Institute on Aging, Intramural Research Program, Baltimore, MD USA

## Abstract

**Background:**

While immune function is known to play a mechanistic role in Alzheimer’s disease (AD), whether immune proteins in peripheral circulation influence the rate of amyloid‐b (Aβ) progression remains unknown.

**Method:**

Using the Baltimore Longitudinal Study of Aging (BLSA; n = 196; mean follow‐up: 5 years/4 scans), we identified immune‐related proteins in plasma (*candidate proteins*) related to rates of change in cortical Aβ levels, as measured by ^11^C‐PiB PET. Along with identifying genetic variants that contributed to candidate protein associations, characterizing their relationships with tau‐PET and changes in ADRD biomarkers (Aβ_42/40_, NfL, GFAP, pTau‐181), and assessing their expression patterns in human microglia, we leveraged data from the Atherosclerosis Risk in Communities (ARIC) study to determine if changes in candidate protein levels precede Ab = β onset (n = 272), and whether they predict 20‐year dementia risk during mid‐life (n = 11,596) and 8‐year dementia risk during late‐life (n = 4,288).

**Result:**

32 immunological proteins in plasma (out of 942) were associated with cortical Ab = β accumulation (**Figure 1A**). Longitudinal changes in a subset of candidate proteins also predicted cortical Aβ accumulation (**Figure 1B**), and the mid‐ to late‐life trajectory of one protein, CAT, was associated with late‐life Aβ‐positive status in ARIC (**Figure 1C**). Genetic variants associated with the plasma abundance of CAT and two additional candidate proteins (CD36 and KRT19) predicted rates of Aβ accumulation (**Figure 2A‐F**). In addition to associations with other AD‐related phenotypes (tau‐PET, plasma AD biomarker changes) (**Figure 2G‐H**), we showed that 31% of candidate proteins were related to mid‐life (20‐year) or late‐life (8‐year) dementia risk in ARIC (**Figure 2I**). Single‐cell RNA‐seq showed a prominent dysregulation of candidate protein expression in homeostatic, interferon‐response and disease‐associated human microglia subtypes (**Figure 3A**). Candidate proteins were enriched for biological processes implicated in AD (**Figure 3B**), susceptible to regulation by key pro‐inflammatory cytokines (**Figure 3C**), displayed robust protein‐interaction networks, showed differential expression in AD brains (especially neurovascular cell types), were externally validated, and are targets of existing therapeutics (**Figure 3D**).

**Conclusion:**

Findings provide insights into how immune proteins in peripheral circulation influence the rate of Aβ accumulation, and identify specific peripheral immune mediators that may contribute to the progression of AD pathophysiology.